# Waist-to-hip ratio better reflect beta-cell function and predicts diabetes risk in adult with overweight or obesity

**DOI:** 10.1080/07853890.2025.2462447

**Published:** 2025-02-08

**Authors:** Na Liu, Bian Wang, Guanxiong Zhang, Minxue Shen, Peng Cheng, Zhanjun Guo, Linhui Zuo, Junya Yang, Min Guo, Min Wang, Zhenqi Liu, Jing Wu

**Affiliations:** ^a^ Department of Endocrinology, Xiangya Hospital, Central South University, Changsha, Hunan, China; ^b^ Hunan Engineering Research Center for Obesity and Its Metabolic Complications, Xiangya Hospital, Central South University, Changsha, Hunan, China; ^c^ Department of Dermatology, Xiangya Hospital, Central South University, Changsha, Hunan, China; ^d^ National Clinical Research Center for Geriatric Disorders, Xiangya Hospital, Central South University, Changsha, Hunan, China; ^e^ Department of Social Medicine and Health Management, Xiangya School of Public Health, Central South University, Changsha, Hunan, China; ^f^ Division of Endocrinology and Metabolism, Department of Medicine, University of Virginia School of Medicine, Charlottesville, VA, USA

**Keywords:** Waist-to-hip ratio, beta-cell function, diabetes

## Abstract

**Objective:**

Controversy remains as to which obesity measures better predict type 2 diabetes (T2D) risk in overweight or obese individuals. The objective of this study is to determine which commonly used obesity measures better reflect beta cell function and predict T2D risk in participants with overweight or obesity and to validate the findings using prospective cohort data.

**Patients and methods:**

Cross-sectional data from the Obesity Clinic of the Xiangya Hospital of the Central South University and prospective cohort from UK Biobank. BMI, waist circumference (WC), waist-to-hip ratio (WHpR), and waist-to-height ratio (WHtR) were measured. Primary outcomes included beta cell function indices in the cross-sectional study and the occurrence of diabetes obtained from UK Biobank data.

**Results:**

One thousand four hundred and ninety-seven participants with overweight or obesity (median age 29 years, 41% males) and 322,023 UK Biobank participants without diabetes at baseline (mean age 56.83 years, 50.4% males) were studied. WHpR had a stronger association with beta cell function and central body fat distribution than the other three obesity measures irrespective of glucometabolic states. WHpR associated positively with diabetes risk in participants using the hazard ratio scale (HR per SD increase of WHpR, 2.311, 95% CI 2.250–2.374).

**Conclusions:**

WHpR is a superior index in reflecting central body obesity, estimating beta cell function, and predicting T2D risk in people with overweight or obesity compared to BMI, WC, and WHtR.

## Introduction

Increased adiposity is a leading risk factor for type 2 diabetes (T2D) and people with obesity have 4–9 times the risk of developing T2D than their non-obese counterparts [1,2]. Imaging studies like dual energy X-ray absorptiometry (DEXA), CT, and MRI can accurately assess fat distribution but they are expensive and inconvenient. Simple measures like body mass index (BMI), waist circumference (WC), waist-to-hip ratio (WHpR), and waist-to-height ratio (WHtR) have been studied to estimate body adiposity and predict the risk of developing T2D in clinical practice and research settings [3–5]. However, it remains controversial as to which measure can better estimate T2D risk and whether WC, WHpR, or WHtR can better predict the incremental risk of T2D beyond BMI alone [6–10].

The glucometabolic status (GMS) of people with obesity varies from normal glucose metabolism (NGM), impaired glucose regulation (IGR), to T2D. While insulin resistance associated with obesity plays a key role in the early stage of T2D development, insufficient insulin secretion due to decreased beta cell function (BCF) is the decisive factor in the final stage of T2D development [11]. The secretory capacity of beta cells in people with obesity typically increases initially to compensate for insulin resistance and then gradually decreases later, leading to further deterioration in glycemic control [12,13]. Data on the relationship between obesity measures and BCF in people with obesity are actually very limited and controversial. Indeed, while BMI was independently associated with BCF in people with NGM [14] or newly diagnosed T2D [15], we have previously shown that, compared to WC and BMI, WHpR is a better anthropometric measure of BCF in people with obesity [16]. Importantly, no study has examined the relationship between these four simple anthropometric indices and BCF in people with overweight or obesity in different GMS. Furthermore, despite the wide use of oral glucose tolerance test (OGTT) in the clinical practice and research settings to evaluate pancreatic β-cell secretory capacity [17], there is a lack of standardization in which commonly used BCF indices, such as the basal insulin secretion index homeostasis model assessment of β-cell function (HOMA-β), the early phase insulin secretion index (e.g. Δ_I30_/Δ_G30_, CIR_30_) and the late phase insulin secretion index (e.g. AUC_I60–120_/AUC_G60–120_, CIR_120_), better reflect BCF in people with obesity and different GMS.

In the current study, we examined the relationship between simple obesity measures and BCF indices separately in humans with overweight or obesity in different GMS. We hypothesized that anthropometric measure that better reflects body fat distribution could better estimate BCF and predict T2D risk.

## Subjects and methods

The current study comprises two parts: Prospective cross-sectional study and prospective Biobank data validation analysis. This research adhered to the ethical guidelines outlined in the Declaration of Helsinki.

### Part I. Prospective cross-sectional study

#### Study protocol

**Figure 1.**
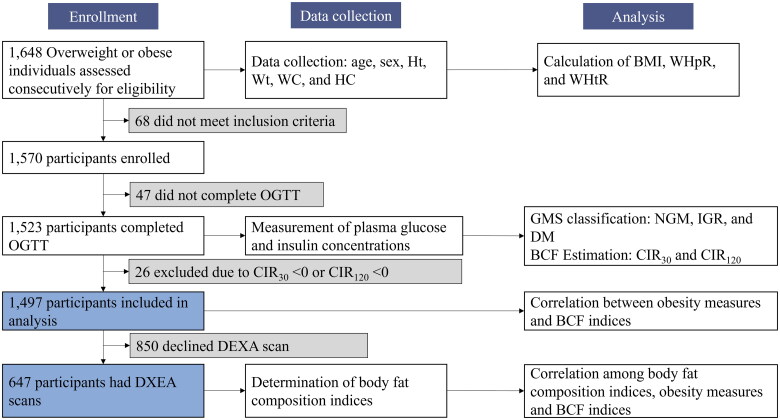
Study flowchart. BCF: beta-cell function; BMI: body mass index; CIR: corrected insulin response; DEXA: dual energy X-ray absorptiometry; DM: diabetes mellitus; GMS: glucometabolic status; Ht: height; IGR: impaired glucose regulation; NGM: normal glucose metabolism; OGTT: oral glucose tolerance test; WC: waist circumference; WHtR: waist-to-height ratio; WHpR: waist-to-hip ratio; Wt: weight.

A total of 1648 individuals with either overweight or obesity (BMI ≥ 24 kg/m^2^ or WC ≥ 85 cm in women or ≥90 cm in men) (age 18–70 years) was consecutively recruited from the Central South University Xiangya Hospital Obesity Clinic between February 2016 and December 2023. Subjects with a known diagnosis of diabetes, with secondary obesity, or who were taking medications known to affect weight in the past 3 months were not offered the study participation. After excluding individuals without a complete anthropometric profile, 1570 participants proceeded to undergo the 75 g OGTT. Of these, 47 participants did not complete the OGTT, and 26 participants had a CIR_30_ or CIR_120_ value of <0. Consequently, these individuals were excluded, leaving a final analysis cohort of 1497 subjects (614 men and 883 women) (Figure 1). All subjects were offered to have body composition determined using DEXA but 850 subjects declined, leaving a total of 647 participants completing the DEXA examination.

The study was approved by the Ethical Committee of Xiangya Hospital of Central South University (Ethics Board Approval Number: 201601029) and all participants gave written informed consent prior to study participation.

#### Data collection

Each subject had a physical examination and all anthropometric measurements were performed using standard methods following an overnight fast. In the subset of subjects who had DEXA scans (Lunar Radiation, Madison, WI, USA), body composition and fat distribution, including trunk fat, arm fat, leg fat, android fat, gynoid fat, android-to-gynoid ratio (A/G), trunk-to-leg fat ratio (T/L), and trunk-to-extremity fat ratio (T/E), were determined.

#### Assessment of glucose metabolism and BCF

All subjects underwent a 75 g OGTT with plasma samples collected at 0 (fasting), 30-, 60-, and 120-min. Plasma concentrations of glucose and insulin were measured at the Xiangya Hospital clinical laboratory. GMS was classified into three groups (NGM, IGR, and T2D) using the WHO 2006 criteria [18].

BCF indices were estimated by modeling glucose and insulin concentrations during the OGTT. While multiple BCF indices have been used to assess BCF, we opted to use the OGTT-based CIR indices, which include the basal insulin secretion index homeostasis model assessment of β-cell function (HOMA-β), the early phase insulin secretion index (e.g. Δ_I30_/Δ_G30_, CIR_30_) and the late phase insulin secretion index (e.g. AUC_I60–120_/AUC_G60–120_, CIR_120_) [19–24] (eTable 1). The performance of the BCF indices in distinguishing abnormal glucose metabolism from NGM was assessed using area under the curve (AUC) (eTable 2). CIR_30_ and CIR_120_ reflect early and late phase insulin secretory indices respectively.

#### Statistical analysis

SPSS version 26.0 (IBM, Chicago, IL, USA) and R-3.6.2 software (https://www.r-project.org) were used for analysis. Normally distributed data are presented as mean ± standard deviation (*SD*) and the skewed data are presented as geometric means with interquartile ranges (IQRs). Differences across different glucometabolic groups were compared in univariable analysis with the use of Kruskal–Wallis test for continuous variables and chi-square test for the categorical variables. Within each group, the Pearson or Spearman’s correlation coefficient and linear regression were used to analyze the correlation among obesity measures, body fat composition, and BCF indices. For correlation analysis, CIR_30_ and CIR_120_ were logarithmically transformed. Multivariate logistic regression was used to estimate the ORs and 95% CIs for the BCF per SD change of obesity measure.

### Part II. Prospective validation analysis

To examine the performance of BMI, WC, WHpR, and WHtR in predicting T2D risk in different human population, we analyzed data from the UK Biobank. After excluding those with existing diabetes at baseline (*n* = 13,673) and BMI < 25 kg/m^2^ (*n* = 166,468), a total of 322,023 participants with overweight or obesity was included in the final analysis. Participants who had self-reported or hospital-diagnosed T2D during follow-up were identified as having developed T2D. We used Cox-PH proportional hazard regression models to separately estimate the associations of each anthropometric measure and the risk of developing T2D after controlling for sex and age, which was reported as hazard ratio (HR) with 95% confidence interval (CI). Unstandardized effects were computed for each 100 bootstrapped samples with 95% CI. Each anthropometric measure was scaled to fit normal distributions centered at 0 with a standard deviation of 1 and performed to estimate the HR per 1−*SD* higher baseline level. The current analyses were carried out under Application Number 101857.

## Results

### General characteristics of part I study participants

The clinical and biochemical characteristics are included in eTable 3. Nearly 60% of the participants were women. Based on the OGTT results, 658 subjects had NGM, 443 had IGR, and 396 were diagnosed with T2D. Subjects with IGR and T2D were older and more insulin resistant (higher homeostasis model assessment of insulin resistance [HOMA-IR]) and had worse BCF (CIR_30_ and CIR_120_) than subjects with NGM. Despite a similar BMI and WC among the three groups, participants with T2D or IGR had significantly higher WHpR and WHtR than individuals with NGM.

### Correlation analysis between CIR_30_ or CIR_120_ and anthropometric measures

**Figure 2.**
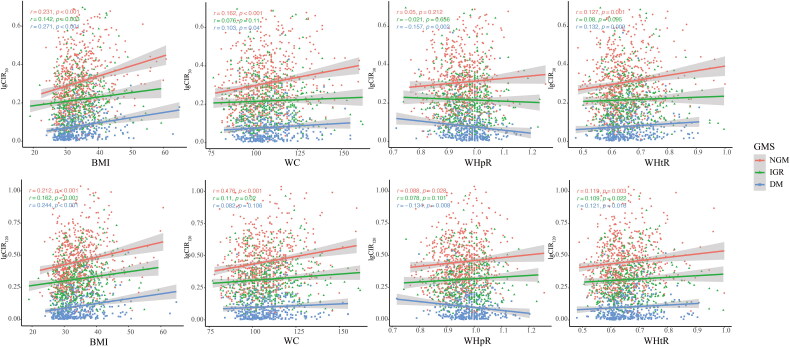
Correlation analysis between BCF indices (CIR30 or CIR120) and anthropometric measures. BMI: body mass index; GMS: glucose metabolism status; NGM: normal glucose metabolism; IGR: impaired glucose regulation; DM: diabetes mellitus; WC: waist circumference; WHtR: waist-to-height ratio; WHpR: waist-to-hip ratio.

Scatter plots showed that BMI and WHtR correlated positively with both CIR_30_ and CIR_120_ across all GMS. WC was positively associated with CIR_30_ and CIR_120_ in the NGM group. WHpR was the only anthropometric measure that was positively associated with CIR_120_ in the NGM group but negatively associated with CIR_120_ in the T2D group (Figure 2 and eTable 4). After adjusting for age, sex, and HOMA-IR, most associations were moderately attenuated but remained statistically significant, and independent associations between WHpR and CIR_30_ (OR per SD, 0.966) or CIR_120_ (OR per SD, 0.943) persisted in the T2D group (Table 1).

**Table 1.** Linear regression association analysis between anthropometric measurements and CIR_30_ or CIR_120_.

GMS	Variable	CIR_30_		CIR_120_	
		OR (95% CI)	*p*-Value	OR (95% CI)	*p*-Value
NGM	BMI	1.052 (0.992, 1.115)	0.090	0.984 (0.868, 1.116)	0.799
	WC	1.041 (0.980, 1.106)	0.188	0.979 (0.861, 1.115)	0.753
	WHpR	1.016 (0.957, 1.079)	0.604	0.916 (0.805, 1.041)	0.178
	WHtR	1.029 (0.973, 1.088)	0.316	0.964 (0.855, 1.087)	0.549
IGR	BMI	1.007 (0.957, 1.059)	0.794	1.001 (0.927, 1.080)	0.987
	WC	0.982 (0.934, 1.033)	0.485	0.994 (0.920, 1.073)	0.868
	WHpR	0.986 (0.937, 1.038)	0.586	1.054 (0.975, 1.140)	0.180
	WHtR	0.993 (0.947, 1.040)	0.754	1.002 (0.932, 1.076)	0.962
DM	BMI	1.003 (0.978, 1.029)	0.801	1.014 (0.978, 1.052)	0.442
	WC	0.992 (0.966, 1.019)	0.539	0.983 (0.946, 1.022)	0.389
	WHpR	0.966 (0.941, 0.992)	**0.010**	0.943 (0.908, 0.979)	**0.002**
	WHtR	0.993 (0.969, 1.018)	0.600	0.991 (0.956, 1.027)	0.608

BMI: body mass index; CIR: corrected insulin response; DM: diabetes mellitus; IGR: impaired glucose regulation; NGM: normal glucose metabolism; WC: waist circumference; WHtR: waist-to-height ratio; WHpR: waist-to-hip ratio.

Odds ratios (95% CIs) per 1−*SD* increment in each anthropometric marker were calculated using logistic regression model after adjusting for age, sex, and HOMA-IR. Bold text indicates statistical significance at *p* < 0.05.

### Correlation among body fat composition, anthropometry, and BCF indices

**Figure 3.**
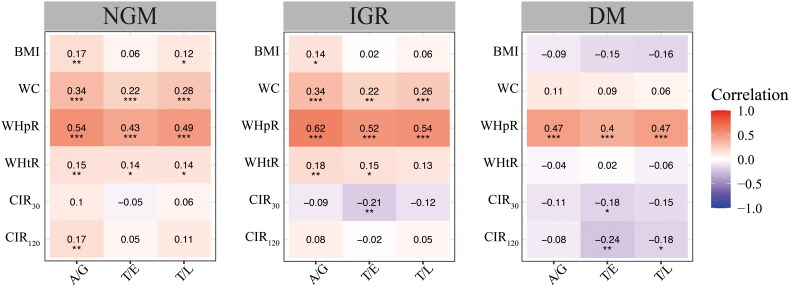
Correlation between anthropometry and body fat distribution indices. A/G: android-to-gynoid ratio; BMI: body mass index; CIR: corrected insulin response; DM: diabetes mellitus; IGR: impaired glucose regulation; NGM: normal glucose metabolism; T/E: trunk-to-extremities fat ratio; T/L: trunk-to-leg fat ratio; WC: waist circumference; WHtR: waist-to-height ratio; WHpR: waist-to-hip ratio. Significance *p*-value two-tails: *p < 0.05, **p < 0.01.

To determine which anthropometric index is better associated with GMS and T2D risk in our study population, we assessed body fat distribution using DEXA scans in 647 subjects. As shown in eTable 5, individuals with T2D had more central fat distribution (i.e. higher T/E, T/L, and A/G ratios) than those with either NGM or IGR and people with IGR and T2DM had less lower extremity fat (gynoid fat and leg fat) compared to their NGM counterparts. Figure 3 shows all four anthropometric measures correlated positively with T/L, T/E, and A/G ratios, which all reflect central body fat distribution, in NGM and IGR group. Compared with the other three obesity indices, WHpR had the strongest correlation with T/L, T/E, and A/G ratios, and was the only obesity measure correlated with all three central body fat distribution in the T2D group. While the associations between body fat compositions and BCF indices varied markedly across all three groups, T/E ratios negatively correlated with CIR_30_ in both IGR group and T2D group, and with CIR_120_ in the T2D group.

### Associations of anthropometric measures with risk of incident T2D

**Figure 4.**
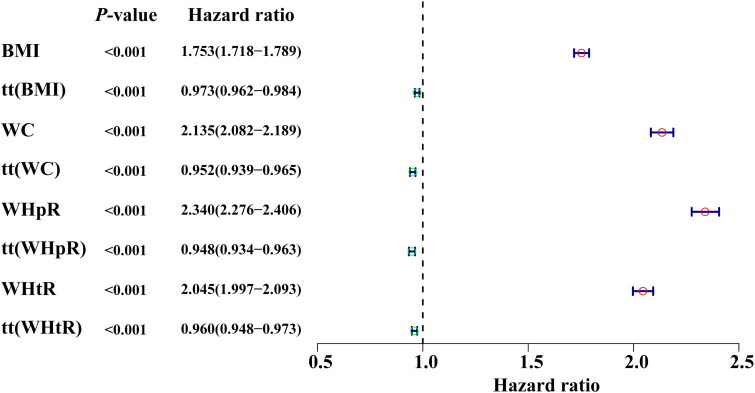
Adjusted odds ratios for risk factors associated with risk of incident T2D among participants with overweight or obesity. BMI: body mass index; WC: waist circumference; WHtR: waist-to-height ratio; WHpR: waist-to-hip ratio. HRs (95% CIs) per 1−*SD* increment in each anthropometric marker were calculated in Cox proportional hazards model after adjusting for age and sex.

Within the UK Biobank cohort, mean (*SD*) age was 56.83 (8.00) years, 50.4% were male, and mean follow-up was 16 (1) years. Among 322,023 participants in the association analyses population, of whom 29,789 developed incident T2D. The proportional hazards assumption was examined by creating a product term of follow-up time and anthropometric measures, and the assumption of proportional hazards was violated for the association between anthropometric measures change during T2D (eTable 6). Therefore, we evaluated these anthropometric indices as a time-dependent covariate on incident T2D (Figure 4). Higher BMI, WC, WHpR, and WHtR were each associated with a greater risk of T2D after adjusting for age and sex. However, on a per 1−*SD* increment basis, WHpR was clearly associated with a stronger risk of T2D than other indices.

## Discussion

The current study strongly suggests that WHpR is superior to BMI, WC, and WHtR in assessing the risk for T2D in humans with overweight and obesity as it more accurately reflects early decline of BCF in those without T2D as well as the decline of late phase insulin secretory capacity in individuals with T2D. A likely explanation is that WHpR better reflects central body fat distribution than other indices. Considering visceral fat accumulation occurs early in the development of obesity, WHpR is thus a better and more sensitive screening tool than BMI, WC, or WHtR in identifying individuals at risk of T2D at much earlier stage and thus enables earlier lifestyle and/or pharmaceutical intervention to delay the onset of T2D.

Multiple anthropometric measures have been used to assess obesity and diabetes risk in clinical practice and research. Among them, BMI is the most widely used but WC is regarded as a more useful predictive risk index for T2D. Our data showed that while BMI, WC, and WHtR each showed a positive trend with BCF in individuals with overweight and obesity across different GMS, WHpR was the only anthropometric measure that positively correlated with BCF in those with NGM, no correlation in those with IGR, and negatively in those with T2D. Multiple linear analysis showed that independent of HOMA-IR, higher WHpR was associated with worse BCF in the T2D group. These results indicate that all four anthropometric parameters could reflect the compensatory increase of BCF in overweight or obesity with NGM, but only WHpR could reflect the changes of BCF from compensatory increase to decompensation in humans with overweight or obesity.

Given that visceral fat affects BCF decline more than BMI [25], and our data show that WHpR is a superior indicator that can better reflect the BCF in individuals with overweight or obesity across the spectrum of GMS, a likely explanation is that WHpR could more accurately reflect visceral fat distribution than other measures. To this end, we quantified body fat distribution using DEXA scans in nearly half of the study population. Our data showed that there were significant differences in fat distribution among different GMS groups, with those having worse GMS showing increased visceral fat. Indeed, WHpR correlated much better with DEXA-derived fat distribution index than WC or WHtR in different GMS groups. The superiority of WHpR over other measures in differentiating GMS is due to (1) intraperitoneal adipose mass being a major contributor to diabetes risk than subcutaneous adipose, typically in the gluteofemoral area, fat depot [26], (2) WC and WHtR not being able to distinguish subcutaneous *vs.* visceral fat depots [27], and (3) WHpR considering the impact of subcutaneous adipose tissue by correcting the WC with hip circumference through the measurement of buttock fat size. This is important as gynoid fat distribution is associated with a better BCF than an android pattern and WHpR can better reflect the relative android/gynoid fat distribution thus BCF than other anthropometric measures. It is important to note that while obesity is a significant risk factor for CKD, in people with end-stage CKD a higher BMI is associated with improved survival, a phenomenon called ‘obesity paradox’. However, BMI does not differentiate among fat tissue, lean tissue, and bone mass. A recent meta-analysis shows that routine use of abdominal obesity measurements in obese CKD patients might allow better risk assessment than using BMI or fat tissue quantity as compared to the overall population, the higher the WHR, the higher the mortality risk [28] Thus, our results could potentially guide obesity management in people with CKD.

Commonly used BCF indices include the basal insulin secretion index and the early and late phase insulin secretion indices. As postprandial hyperglycemia occurs earlier than fasting hyperglycemia during T2D development [29], it is important to assess all phases of insulin secretory capacity in predicting the risk of T2D. Indeed, previous study has shown that insulin response at 30 min after the oral glucose load is a major factor affecting glucose homeostasis [30]. We compared the utility of different BCF indices in differentiating different GMS, and CIR_120_ and CIR_30_ ranked 1st and 2nd in distinguishing T2D from NGM and IGR, respectively. The CIR indices provide more comprehensive assessment of BCF than other indices, such as I/G and ΔI/ΔG [21], and have been widely used to evaluate pancreatic β-cell secretory capacity in the clinical practice and research settings [17,31,32]. In our study, a dramatic decrease in CIR_30_ and CIR_120_ occurred in the IGR state and a loss in postprandial glycemic control preceded glycemic deterioration in the fasting state with disease progression to IGR and T2D. This is consistent with a prior study, conducted in Iraq and Sweden, demonstrating that CIR predicted T2D onset independent of BMI and WHpR [33]. Thus, CIR_30_ and CIR_120_ are more sensitive BCF indices in distinguishing GMS in individuals with overweight or obesity, particularly early in the disease progression.

Our part 1 study is a cross-sectional study that only captures data at a single point in time, making it impossible to determine cause-and-effect relationships between the variables, and is also prone to selection bias. To overcome the limitation of our prospective population-based study in only one ethnic population, we examined whether our study findings can be extrapolated to other ethnic groups by comparing the ability of each of the four anthropometric measures in predicting the development of T2D in individuals with obesity using data derived from UK Biobank (i.e. different population at different time point). The UK Biobank data included 322,023 participants who were free of T2D at baseline and had longitudinal follow-up for the development of diabetes and thus serves as an excellent resource to corroborate our study findings. While the UK Biobank data analysis suggested that all four anthropometric measures were associated with the risk of developing T2D, higher WHpR was associated with much stronger risk of T2D, with no overlapping of 95% CI of HR values among WHpR and other three measurements. Thus, analysis of UK Biobank data which included participants from multiple ethnic backgrounds, support our conclusion that WHpR is a superior index in predicting T2D risk.

## Conclusions

In our study, we found that WHpR is a superior index in reflecting visceral fat distribution, estimating BCF, and predicting T2D risk in population with overweight or obesity. Visceral fat plays a critical role in the development of insulin resistance and T2D by attracting inflammatory immune cells and secreting pro-inflammatory cytokines and adipokines [34,35]. However, the direct measurement of visceral fat requires advanced imaging technologies, which are often impractical for routine clinical practice. Our findings demonstrate that WHpR has the strongest association with visceral fat distribution, making it an ideal surrogate marker for screening. The widespread use of WHpR in clinical practice could enable clinicians to identify individuals at higher risk of developing T2D, facilitating early interventions to prevent or delay the progression of the disease.

## Supplementary Material

Supplementary File.docx

## Data Availability

The data that support the findings of this study are available on request from the corresponding author Jing Wu. The data are not publicly available due to their containing information that could compromise the privacy of research participants.
