# Supplementary material for: Waist-to-hip ratio better reflect beta-cell function and predicts diabetes risk in adult with overweight or obesity
Source: Ann Med. 2025 Feb 8;57(1):2462447. doi: 10.1080/07853890.2025.2462447 (PMC11809193; doi:10.1080/07853890.2025.2462447)
Supplement: Supplementary File.docx [file IANN_A_2462447_SM8028.docx]

**eTable 1. BCF indices based on OGTT**

| BCF index | Formula |
| --- | --- |
| HOMA-β | 20 × I0 / (G_0_ - 3.5) |
| CIR_30_ | I_30_ × 100 / [G_30_ × 18 × (G_30_ × 18 - 70)] |
| CIR_120_ | I_120_ × 100 / [G_120_ × 18 × (G_120_ × 18 - 70)] |
| 1^st^ PH | 1283+0.305 × I_30_-138.7 × G_30­_ + 0.629 × I_0_ |
| 2^nd^ PH | 287 + 0.0694 × I_30_ – 26.07 × G_30_ + 0.1538 × I_0_ |
| IGI | (I_30_ - I_0_) / (G_30_ - G_0_) / 18 |
| MBCI | (G_0_ × I_0_) / (G_60_ _­_+ G_120_-7.0) |
| I_AUC_/ G_AUC_ | [(30-0) × (I_0_+I_30_)/2] + [(60-30) × (I_30_+I_60_) / 2] + [(120-60) × (I_60_+I_120_)/2)] / [(30-0) × (G_0_+G_30_) /2] + [(60-30) × (G_30_+G_60_)/2] + [(120-60) × (G_60_ + G_120_)/2] |

Abbreviations: ^1st^ PH, First-phase insulin release; 2^nd^ PH, Second-phase insulin release; AUC, area under the curve, calculated using the trapezoidal method; CIR, corrected insulin response; IGI, insulinogenic index; I, plasma insulin concentrations (μIU/mL); G, plasma glucose concentrations (mmol/L); MBCI: modified beta cell function index.

**eTable 2. Ranking of BCF indices in distinguishing different glucose metabolic states using the ROC AUC method**

| **BCF index** | NGM vs IGR | Rank | IGR vs DM | Rank | NGM vs DM | Rank |
| --- | --- | --- | --- | --- | --- | --- |
| HOMA-β | 0.550 | 7 | 0.592 | 7 | 0.627 | 8 |
| CIR_30_ | 0.668 | 4 | 0.667 | 2 | 0.789 | 2 |
| CIR_120_ | 0.711 | 2 | 0.689 | 1 | 0.838 | 1 |
| 1^st^ PH | 0.666 | 5 | 0.648 | 4 | 0.782 | 3 |
| 2^nd^ PH | 0.668 | 3 | 0.651 | 3 | 0.782 | 4 |
| IGI | 0.633 | 6 | 0.604 | 5 | 0.729 | 5 |
| MBCI | 0.733 | 1 | 0.448 | 8 | 0.644 | 6 |
| I_AUC_/G_AUC_ | 0.545 | 8 | 0.603 | 6 | 0.642 | 7 |

Abbreviations: ^1st^ PH, First-phase insulin release; 2^nd^ PH, Second-phase insulin release; AUC, area under the curve, calculated using the trapezoidal method; CIR, corrected insulin response; IGI, insulinogenic index; I, plasma insulin concentrations (μIU/mL); G, plasma glucose concentrations (mmol/L); MBCI: modified beta cell function index.

**eTable 3. Clinical and biochemical characteristics of the individuals with overweight or obesity according to GMS**

|  | NGM (*n*=658) | IGR (*n*=443) | DM (*n*=396) | *P*-value |
| --- | --- | --- | --- | --- |
| Age, years | 26 (21-32) | 30 (25-37)^b^ | 35 (29-47)^bc^ | <0.001 |
| Sex, female (%) | 388 (59%) | 296(66.8%) | 199 (50.3%)^bc^ | <0.001^a^ |
| BMI, kg/m^2^ | 33.19 (30.11-37.16) | 33.64 (30.22-37.77) | 32.30 (29.56-36.67) | 0.063 |
| WC, cm | 105.00 (97.00-114.13) | 106.50 (98.00-116.00) | 106.00 (99.00-114.00) | 0.190 |
| WHpR | 0.95 (0.90-1.00) | 0.97 (0.92-1.02)^b^ | 0.98 (0.94-1.02)^b^ | <0.001 |
| WHtR | 0.64 (0.60-0.70) | 0.65 (0.61-0.71)^b^ | 0.65 (0.61-0.70) | 0.001 |
| HOMA-IR | 4.06 (2.93-5.95) | 5.10 (3.38-7.46)^b^ | 7.07 (4.63-11.41)^bc^ | <0.001 |
| CIR_30_ | 0.97 (0.61-1.59) | 0.59 (0.35-0.92)^b^ | 0.14 (0.06-0.27)^bc^ | <0.001 |
| CIR_120_ | 1.65 (1.09-2.79) | 0.98 (0.58-1.53)^b^ | 0.16 (0.06-0.40)^bc^ | <0.001 |

Abbreviation: BMI, body mass index; CIR, corrected insulin response; DM, diabetes mellitus; HOMA-IR, homeostasis model assessment of insulin resistance; IGR, impaired glucose regulation; NGM, normal glucose metabolism; WC, waist circumference; WHtR, waist-to-height ratio; WHpR, waist-to-hip ratio.

Values are expressed as median (IQR). *P*-value of Kruskal–Wallis test.

^a^*P*-value of chi-square test.

^b^Statistically significant difference vs NGM, *P*-value < 0.05.

^c^Statistically significant difference vs IGR, *P*-value < 0.05.

HRs (95% CIs) per 1-SD increment in each anthropometric marker were calculated in Cox proportional hazards model.

**eTable 4. Correlation analysis between CIR_30_ or CIR_120_ and clinical and biochemical characteristics**

|  | CIR_30_ | | | | | | | | | | CIR_120_ | | | | | | | |
| --- | --- | --- | --- | --- | --- | --- | --- | --- | --- | --- | --- | --- | --- | --- | --- | --- | --- | --- |
|  | NGM (*n*=628) | | IGR (*n*=441) | | | DM (*n*=395) | | | NGM (*n*=628) | | | | IGR (*n*=441) | | | DM (*n*=395) | | |
|  | r | *P* | | r | *P* | | r | *P* | | r | | *P* | | r | *P* | | r | *P* |
| Age | -0.294 | <0.001 | | -0.351 | <0.001 | | -0.302 | <0.001 | | -0.228 | | <0.001 | | -0.352 | <0.001 | | -0.296 | <0.001 |
| Sex | -0.077 | 0.055 | | 0.060 | 0.212 | | 0.193 | <0.001 | | -0.163 | | <0.001 | | -0.101 | 0.035 | | 0.193 | <0.001 |
| HOMA-IR | 0.422 | <0.001 | | 0.480 | <0.001 | | 0.251 | <0.001 | | 0.506 | | <0.001 | | 0.609 | <0.001 | | 0.231 | <0.001 |

Abbreviation: HOMA-IR, homeostasis model assessment of insulin resistance.

**eTable 5. Baseline characteristics of participants with DEXA scans**

|  | NGM (*n*=301) | IGR (*n*=217) | DM (*n*=129) | *p*-value |
| --- | --- | --- | --- | --- |
| Age, years | 26 (21-32) | 30 (24-35) ^a^ | 32 (25-41) ^a^ | <0.001 |
| Sex, female (%) | 168 (55.8%) | 142 (65.4%)^a^ | 73 (56.6%)^b^ | 0.002 |
| BMI, kg/m^2^ | 33.48 (30.48-37.45) | 34.25 (30.62-37.68) | 33.96 (30.60-38.74) | 0.742 |
| WC, cm | 106.00 (98.75-115.00) | 107.00 (98.00-116.00) | 108.00 (99.00-116.25) | 0.892 |
| WHpR | 0.96 (0.92-1.00) | 0.96 (0.91-1.02) | 0.97 (0.64-1.01) | 0.168 |
| WHtR | 0.64 (0.61-0.69) | 0.65 (0.61-0.71) | 0.66 (0.61-0.70) | 0.198 |
| HOMA-IR | 4.23 (3.05-5.92) | 5.90 (3.80-8.16) ^a^ | 8.84 (5.45-14.85) ^ab^ | <0.001 |
| CIR_30_ | 0.97 (0.58-1.51) | 0.62 (0.37-0.92) ^a^ | 0.17 (0.07-0.34) ^ab^ | <0.001 |
| CIR_120_ | 1.67 (1.08-2.62) | 1.00 (0.62-1.64) ^a^ | 0.23 (0.07-0.34) ^ab^ | <0.001 |
| Android | 45.98±4.98 | 46.14±5.00 | 45.08±5.66 | 0.154 |
| Gynoid | 39.10 (4.80) | 38.50 (4.55) | 36.70 (3.60) ^ab^ | 0.011 |
| Trunk | 42.71±5.26 | 42.91±5.27 | 42.03±5.84 | 0.326 |
| Arm | 47.10 (6.05) | 47.90 (5.30) | 46.05 (8.20) | 0.111 |
| Leg | 38.75 (4.78) | 38.85 (4.58) | 36.20 (4.98) ^ab^ | 0.011 |
| A/G | 1.19 (1.09-1.29) | 1.20 (1.13-1.30) | 1.23 (1.14-1.34) ^a^ | 0.029 |
| T/L | 1.11 (1.02-1.21) | 1.12 (1.05-1.26) | 1.19 (1.06-1.30) ^a^ | 0.002 |
| T/E | 1.21 (1.04-1.39) | 1.24 (1.11-1.40) | 1.33 (1.15-1.51) ^a^ | <0.001 |

Abbreviation: A/G, android-to-gynoid ratio; BMI, body mass index; CIR, corrected insulin response; DM, diabetes mellitus; HOMA-IR, homeostasis model assessment of insulin resistance; IGR, impaired glucose regulation; NGM, normal glucose metabolism; T/E, trunk-to-extremities fat ratio; T/L, trunk-to-leg fat ratio; WC, waist circumference; WHtR, waist-to-height ratio; WHpR, waist-to-hip ratio.

Values are expressed as median (IQR). *P*-value of analysis of Kruskal–Wallis test.

^a^Statistically significant difference vs NGM, *P*-value < 0.05.

^b^Statistically significant difference vs IGR, *P*-value < 0.05.

**eTable 6. Estimated Cox models and the tests of the PH assumption**

| Variables | HR (95% CI) | PH assumption (*P-*value) |
| --- | --- | --- |
| BMI (per 1 SD) | 1.636 (1.025-2.247) | <0.001 |
| WC (per 1 SD) | 1.907 (1.383-2.431) | <0.001 |
| WHpR (per 1 SD) | 2.094 (1.617-2.571) | <0.001 |
| WHtR (per 1 SD) | 1.854 (1.850-1.858) | <0.001 |

Abbreviation: BMI, body mass index; WC, waist circumference; WHtR, waist-to-height ratio; WHpR, waist-to-hip ratio; PH, proportional hazards.

HRs (95% CIs) per 1-SD increment in each anthropometric marker were calculated in Cox proportional hazards model.
